# Mindfulness in trauma recovery: applications, insights, and future directions

**DOI:** 10.3389/fpsyg.2025.1700047

**Published:** 2025-11-06

**Authors:** Brian Chopko, Michael Mantzios, Konstantinos Papazoglou, Richard Adams

**Affiliations:** 1 Kent State University at Stark, North Canton, OH, United States; 2 Birmingham City University, Birmingham, United Kingdom; 3 ProWellness Inc., Psychology Professional Corporation, Woodbridge, ON, Canada; 4 Kent State University, Kent, OH, United States

**Keywords:** mindfulness, trauma, psychological health, first responders, applications

## Introduction

First introduced into the Western clinical academic literature in the early 1980s (Kabat-Zinn, 1982; Brown and Ryan, 2003), mindfulness-based interventions and programs have seen substantial growth in recent years as a means to improve health and wellbeing (Baroni et al., 2018; Brown and Ryan, 2003; Chopko et al., 2018; Mantzios and Giannou, 2014; Mantzios et al., 2023). In recognition of the increasing scholarly and clinical attention to this concept, Frontiers in Psychology established a Mindfulness section in 2022. In this article, we briefly review the conceptualization of mindfulness and its different facets, done in the context of the section's third anniversary. Furthermore, we discuss how mindfulness, both as a disposition and the result of training, can improve the health and wellbeing of people in specific occupations or populations facing potentially traumatic and stressful life events.

## What is mindfulness?

As several writers note, mindfulness, as an aspect of consciousness, has typically been defined as “the state of being attentive to and aware of what is taking place in the present” (Brown and Ryan, 2003: p. 822). Further, it has been concerned with the mental state of non-judging, openness, curiosity, and acceptance of internal functioning and emotions and external situations emerging from current ongoing experiences (Baroni et al., 2018; Choi et al., 2021). However, these writers have also noted that mindfulness has been defined in several ways, with some research focusing on engagement with experiences and others examining mindfulness as relief from stress and a means to achieve resilience and psychological health, leading one writer to ask, “What do people mean when they talk about mindfulness?” (Choi et al., 2021). It has been used in research as both an object to be explained and as an intervention to make people's lives better. Thus, most scholars argue that we need much more theoretical and conceptual clarity of this concept as both a dependent and independent variable (Siegel et al., 2009; Singh, 2010). Future attempts to redefine mindfulness should be made within a broader historical lens. That is, mindfulness has roots in Eastern philosophies, most notably Buddhism, which involves complex meanings not easily translated and sometimes mistranslated by Western culture (Bodhi, 2011; Wilson, 2014). This contributes to the definition of mindfulness lacking consensus and clarity in recent literature (Van Dam et al., 2018).

## Mindfulness as a dependent variable is multi-faceted

One development of the term has been to move away from conceptualizing mindfulness as a unitary concept (e.g., Brown and Ryan, 2003) and toward a multifaceted one. Bishop et al. (2004), for example, propose a two-component definition of mindfulness. First, self-regulation of attention brings awareness to current experience, “observing and attending to the changing field of thoughts, feelings, and sensations from moment to moment,” while orientation to experience is a commitment to experiences characterized by “curiosity, openness, and acceptance” (Bishop et al., 2004: p. 232).

As another example, Baer et al. (2008) argue for a five-facet definition (Observing, Describing, Acting with Awareness, Nonjudging of Inner Experience, and Nonreactivity in Inner Experience). Observing focuses on noticing or attending to internal and external experiences, which could be physical sensations, sights, sounds, cognitions, and emotions. Describing refers to giving internal experiences specific labels. Acting with awareness includes paying attention to your activities “in the here and now,” in contrast to behaving mechanically (automatic pilot). Non-judging of inner experience is taking a non-evaluative stance toward thoughts and feelings. Lastly, nonreactivity to inner experience allows thoughts and feelings to come and go, without getting carried away by them (Baer et al., 2008).

Both conceptualizations of mindfulness as multifaceted have led to significant research programs (e.g., Bowlin and Baer, 2012; Carpenter et al., 2019; Keng et al., 2011; Shapiro et al., 2006; Lubbers et al., 2024). These studies differ from early research that have examined mindfulness with a narrower, unitary conceptualization of this concept. Definitions of mindfulness from Buddhist tradition involve three core elements including memory and remembrance, present-moment awareness, and ethics (Carpenter et al., 2019). Contemporary definitions and early studies in modern psychology and health tended to focus on present-moment awareness, a simple form of the Eastern tradition (Chems-Maarif et al., 2025). Thus, a broader refinement of the construct of mindfulness is ongoing and needed.

## Mindfulness as an independent variable

When examining mindfulness as an independent variable that can promote psychological health, studies have suggested that people often are mindful as a consequence of their socialization or experiences with others (i.e., dispositional mindfulness), but that people can also be trained in mindfulness techniques (Chopko et al., 2018; Tang et al., 2019; Tomlinson et al., 2018). Researchers, however, have noted that mindfulness may be non-monotonic. That is, specific aspects of mindfulness that are above or below certain levels may be counter-productive and result in poor health outcomes (Britton, 2019). Furthermore, consider that different facets of dispositional mindfulness (e.g., acting with awareness, nonjudgmental acceptance, describing) have been associated with improved health outcomes such as lower posttraumatic stress disorder (PTSD) symptoms. An exception to these facets, however, is mindful observing. That is, greater mindful observing has been associated with higher levels of symptoms in disorders such as PTSD (e.g., Chopko et al., 2022). Additionally, mindful observing has also been found to have no association with other disorders such as major depressive disorder (MDD) when the other facets were negatively related (e.g., Soysa and Wilcomb, 2015). As a result, a better understanding of mindful observing is needed, including the reasons for the positive or no relationship with health symptoms as well as any underlying causal mechanisms that may exist.

Various explanations have been put forth to explain the positive relationship between mindful observing and health symptoms. For instance, Raphiphatthana et al. (2016) postulated that observing behaviors may be the result of a hypervigilant state, which is focused on detecting perceived threat in trauma-exposed people. This possibility also has implications for mindfulness-based interventions. For example, it has been suggested that the facilitation of mindful observing has the potential to exacerbate anxiety symptoms for those experiencing anxiety-related disorders (Desrosiers et al., 2013). An important distinction should be made in that it is likely not a matter of what we observe, but rather, how we mindfully observe (e.g., in a non-judgmental and compassionate manner) that distinguishes between healthy and unhealthy outcomes.

## Does mindful observing promote wellbeing?

Various measures of mindfulness exist that assess the observing facet (Baer et al., 2022, 2008). The construct validity of these measures, however, has been questioned (Aguado et al., 2015). Some researchers state that the observing of emotions is critical for psychological wellbeing. Not all mindfulness assessments, however, measure the observing of emotions. As a result, it has been recommended that mindfulness measures assess emotion-focused observing (Rudkin et al., 2018). Also consider that Höfling et al. (2011), in developing a short version of the Kentucky Inventory of Mindfulness Skills, found two different factors regarding mindful observing. Specifically, the differences were based on whether the stimuli being observed were internal (e.g., noticing body sensations while walking) or external (e.g., paying attention to sounds).

Moskow-Diamond et al. (2025) found that increased anxiety sensitivity was related to greater internal observing scores and lower external observing levels. Furthermore, researchers have also distinguished between judgmentally observing and non-judgmentally observing. Höfling et al. (2011) reported that judgmentally observing was related to worse health outcomes on assessments measuring variables such as worry. Höfling et al. recommend that mindfulness-based interventions for those prone to judgmentally observing should focus on non-judgement and self-compassion, especially for those experiencing disorders such as MDD.

Collectively, explanations are rational and fall under two main categories. First, for individuals with PTSD or anxiety-related disorders, observing may promote hypervigilance, over-identification, or rumination, and in such cases, peaceful observing may blur with anxious observing, which can translate into obsessive monitoring of internal distress and environmental threats. Second, while some FFMQ items are benign (e.g., “wind in my hair or sun on my face”), others focus on internal sensations and may capture distressing thoughts and emotions. For example, items like “how foods and drinks affect my thoughts, bodily sensations, and emotions” or “how my emotions affect my thoughts and behavior” may resonate more strongly and painfully with those who have experienced trauma and have different motivations to be observant. For people struggling with eating disorders (e.g., binge eating, anorexia, orthorexia), if their observation is done without acceptance or kindness, it leaves the door open to the possibility that they are observing while simultaneously criticizing themselves, which has been highlighted as a primary problem within mindful eating psychometric measurements and interventions (Mantzios, 2023). The range of populations to which this inconsistency (i.e., internal vs. external, and judgmental vs. non-judgmental, observation) may apply is considerably broad. For instance, adolescence is a time marked by significant brain changes and increased susceptibility to stress, anxiety, and depression. Similarly, chronic conditions, such as cystic fibrosis, diabetes, asthma, cardiovascular disease, or autoimmune disorders may equally exhibit unique vulnerabilities. While chronic illness is not a trauma, it can be a *traumatic stressor*, especially when it is life-altering. For instance, in cystic fibrosis and asthma, internal observation of the breath may be tense, as the act of observing respiration can be a constant reminder of physical dysfunction and decline, and proposes clear implications for mindfulness practices for such populations.

## Mindfulness interventions to promote wellbeing

As a clinical tool, mindfulness interventions have been crucial in helping individuals who present with different types of disorders or symptomatology. Mindfulness is often incorporated as part of other psychological approaches such as Cognitive-Behavioral and Dialectical-Behavioral. Thoughts focused on the mindful observation of stimuli and the resulting responses of the individual may be explored clinically. Additionally, mindfulness may be implemented in a way that assists one to be more aware of their own experiences and symptoms, as well as to compassionately attempt to address those reactions in the therapeutic context. Thus, it would be the first step toward a genuine awareness and decision-making to address current issues and work toward managing symptoms (Lang, 2017; To and Schuman-Olivier, 2024; Treleaven, 2018). On the other hand, awareness of the here and now and one's own experiences and reactions may also mean identifying certain strengths and virtues that the person was unaware of. Such a realization may also function as a vehicle toward addressing issues in the clinical context.

## Future research on mindfulness

The current state of mindfulness research highlights the need for future research to further explore which aspects of mindfulness are salutogenic and if any may be counterproductive. Specifically, a better understanding of mindful observing and the impact on health outcomes is needed. At this moment, it is crucial to determine which specific aspects of mindfulness are beneficial and for whom, ultimately leading to the development of more targeted and safer interventions. To clarify, health and wellness outcomes may depend on the method of instruction as much as the person being instructed on mindfulness techniques. As a result, the tailoring of mindfulness interventions to meet the needs of the individual should be a focus of attention. Lastly, mindfulness research on adolescents and other special populations is also imperative to maximize the benefits of this therapeutic approach.
